# GTSE1 expression represses apoptotic signaling and confers cisplatin resistance in gastric cancer cells

**DOI:** 10.1186/s12885-015-1550-0

**Published:** 2015-07-25

**Authors:** Vinod Vijay Subhash, Shi Hui Tan, Woei Loon Tan, Mei Shi Yeo, Chen Xie, Foong Ying Wong, Zee Ying Kiat, Robert Lim, Wei Peng Yong

**Affiliations:** 1 Cancer Science Institute of Singapore, National University of Singapore, Singapore, Singapore; 2 Department of Haematology-Oncology, National University Hospital of Singapore, Singapore, Singapore

**Keywords:** GTSE1, Drug resistance, Cisplatin, Apoptosis

## Abstract

**Background:**

Platinum based therapy is commonly used in the treatment of advanced gastric cancer. However, resistance to chemotherapy is a major challenge that causes marked variation in individual response rate and survival rate. In this study, we aimed to identify the expression of GTSE1 and its correlation with cisplatin resistance in gastric cancer cells.

**Methods:**

Methylation profiling was carried out in tissue samples from gastric cancer patients before undergoing neoadjuvent therapy using docetaxel, cisplatin and 5FU (DCX) and in gastric cancer cell lines. The correlation between GTSE1 expression and methylation in gastric cancer cells was determined by RT-PCR and MSP respectively. GTSE1 expression was knocked-down using shRNA’s and its effects on cisplatin cytotoxicity and cell survival were detected by MTS, proliferation and clonogenic survival assays. Additionally, the effect of GTSE1 knock down in drug induced apoptosis was determined by western blotting and apoptosis assays.

**Results:**

GTSE1 exhibited a differential methylation index in gastric cancer patients and in cell lines that correlated with DCX treatment response and cisplatin sensitivity, respectively. *In-vitro*, GTSE1 expression showed a direct correlation with hypomethylation. Interestingly, Cisplatin treatment induced a dose dependent up regulation as well as nuclear translocation of GTSE1 expression in gastric cancer cells. Knock down of GTSE1 enhanced cisplatin cytotoxity and led to a significant reduction in cell proliferation and clonogenic survival. Also, loss of GTSE1 expression caused a significant increase in P53 mediated apoptosis in cisplatin treated cells.

**Conclusion:**

Our study identifies GTSE1 as a biomarker for cisplatin resistance in gastric cancer cells. This study also suggests the repressive role of GTSE1 in cisplatin induced apoptosis and signifies its potential utility as a therapeutic target for better clinical management of gastric cancer patients.

**Electronic supplementary material:**

The online version of this article (doi:10.1186/s12885-015-1550-0) contains supplementary material, which is available to authorized users.

## Background

Despite of significant advances in therapeutic strategies and a decline in incidence over the last few decades, gastric cancer still remains as the second most leading cause of cancer-related mortality worldwide [1, 2]. Currently, surgery represents gold standard for the treatment of gastric cancer without distant metastasis. However, chemotherapy appears to be a useful option in the treatment of advanced gastric cancer, with a modest but real survival benefit [3]. Platinum based chemotherapy containing cisplatin and oxaliplatin were shown to have promising results with similar response rate and progression-free survival (Cunningham *et al.*; 2008). Although platinum based therapy is highly active in gastric cancer, a marked individual variation in response rate (RR) and survival rate is seen among patients undergoing treatment. In order to better control the local relapse and increase in survival time of advanced patients, the role of neoadjuvant chemotherapy (NAC) is currently being investigated with different protocols. Multiple phase II and phase III trials utilizing docetaxel, cisplatin and 5-FU (DCX) have shown this combination to be highly effective, particularly in advanced gastric carcinoma [4, 5]. Albeit these advances, the appearance of drug resistance limits the effectiveness of cancer chemotherapy and poses a major impediment in clinical treatment [6]. Earlier studies have revealed the major mechanisms underlying resistance that include reduced uptake and/or increased efflux and enhanced DNA repair [7, 8]. As tumors are highly adaptable, drug resistance can also be induced by the activation of survival signaling pathways and the inactivation of downstream death signaling pathways [9]. Additionally, epigenetic changes, changes in the molecular phenotype and the influence of the local tumor microenvironment, could also play contributory roles in chemoresistance [10]. Hence, elucidating the mechanism underlying the sensitivity and resistance to chemotherapy is critical to develop a more personalized approach towards treatment of gastric cancer.

Human GTSE1 (G2 and S phase expressed-1) is expressed specifically during G2 and S phases of the cell cycle, and is localized mainly in the cytoplasm, associated with microtubules [11]. GTSE1 is cell cycle regulated and becomes phosphorylated in mitosis and markedly reduced in G_1_ phase of cell cycle [12]. Over expression of GTSE1 results in a delay of the G_2_ to M phase transition [13]. The protein is reported to shuttle between the cytoplasm and nucleus, however it gets stabilized in the nucleus following DNA damage. Once in the nucleus, GTSE1 acts as a negative regulator of p53 expression where it binds and relocalizes p53 to the cytoplasm to undergo degradation [14]. Consequentially, the DNA damage induced trans-activation of p53 is inhibited, thus affecting p53 induced apoptosis [14, 15]. In the absence of DNA damage, GTSE1 has been reported to localize to the interphase microtubule networks where it exists in association with clathrin-containing complexes [16, 17]. Tian *et al.* (2011) have shown that GTSE1 is up-regulated in lung cancer tissues compared to the adjacent normal tissues, especially in adenocarcinoma and squamous cell carcinoma. Of interest, a more than two-fold increase in GTSE1 expression was shown in myeloma cells after cisplatin treatment, suggesting a mechanism of clinically acquired drug resistance [18].

This study explored the expression, cellular localization and functional significance of GTSE1 in gastric cancer. GTSE1 methylation was found to be associated with better treatment response to DCX- chemotherapy in gastric cancer patients. A correlation between GTSE1 expression and cisplatin cytotoxicity is suggested here, as cisplatin treatment induced a dose dependent up regulation of GTSE1 in gastric cancer cells. This increase in expression was seen associated with a change in cellular localization as well. Intriguingly, loss of GTSE1 expression contributed to enhanced cisplatin sensitivity and p53 induced apoptotic signaling in gastric cancer cells. Taken together, by identifying the regulatory role of GTSE1 in cisplatin sensitivity and drug induced apoptosis, this study signifies the potential implications of GTSE1 as a biomarker for cisplatin resistance in gastric cancer. Moreover, our study presents an additional candidate for personalised molecular targeted therapy that could overcome cisplatin resistance and thereby attempts to improve the therapeutic index of this compound in clinical applications.

## Methods

### Analysis of microarray datasets

Two independent microarray datasets (Gastric cancer: a, GSE13911; b, GSE27242) of pair wise tumor tissues and adjacent normal tissues were retrieved from www.oncomine.org and the mRNA expression level of GTSE1 was investigated. A total of 169 and 69 samples were analysed in GSE27242 and GSE27242 respectively.

### Patient recruitment and study design

21 consecutive patients with locally advanced [AJCC TNM (T3/4 or N+ M0)] histologically-proven gastric or esophagogastric adenocarcinoma with no evidence of distant metastases, or locally advanced inoperable disease, as evaluated by computed tomography (CT), chest radiography, ultrasonography, or laparoscopy were included in the study. Pre-treatment characteristics of patients are mentioned in Table 1. DCX combination was administered in a 21-day cycle for three cycles before surgery. The first cohort of 10 patients received intravenous docetaxel 35 mg/m^2^, intravenous cisplatin 35 mg/m^2^ on day 1 and day 8, with oral capecitabine 750 mg/m^2^ twice daily from day 1 to day 14. A subsequent cohort of another 11 patients had dose modifications to docetaxel 30 mg/m2, cisplatin 30 mg/m^2^ and capecitabine 700 mg/ m^2^ due to high rates of diarrhea in the first cohort of 10 patients Preoperative radiological response was evaluable in 17 patients after two cycles of chemotherapy (Additional file 1: Figure S1). GTSE1 methylation was determined in 19 patients prior to neo-adjuvent chemotherapy. A total of 14 patients underwent curative surgery and the surgical details are mentioned in Additional file 1: Table S1. Seven patients did not undergo surgery with three having withdrawn consent and two declined surgery after completing neoadjuvant chemotherapy and two had disease progression. Eleven patients underwent post treatment pathological assessment. The median follow-up was 25 (23–27) months after surgery and patients were classified as responders or non-responders by the radiological response (Responders: complete response or near-complete response; Non-responders: partial response or others). The median follow-up was 25 (23–27) months after surgery and patients were classified as responders or non-responders by the radiological response (Responders: complete response or near-complete response; Non-responders: partial response or others). The protocol was reviewed and approved by the institution’s review board and informed consent was obtained from each patient. Ethical approval was obtained from NHG (National Health Group) Domain Specific Review Board, Singapore.

### Genomic methylation profiling

Genomic DNA from primary gastric tissues (11) and cell lines (39) were bisulphite-modified using EZ DNA methylation kit (Zymo Research). The modified samples were profiled in infinium 27 K methylation array (Illumina). DNA methylation levels for each CpG site were computed by Genome Studio software as the ratio of methylated intensity to the sum of methylated and unmethylated signal intensities.

### Cell culture and drug treatment

AZ521 and OCUM-1 were obtained from Japanese Collection of Research Bioresources, Japan. SNU610 and SNU719 were purchased from Korean Cell Line Bank, Korea. All the cell lines were cultured at 37 °C in a humidified atmosphere containing 5 % CO_2_ and maintained in RPMI 1640 medium (Gibco), containing 10 % heat inactivated fetal bovine serum (Gibco) and 1 % penicillin/streptomycin (Gibco). For dose response studies, the cell lines were treated with cisplatin at IC50 concentration.

### Cell Synchronization

Cells were synchronized at the G_1_/S border by treating with 2.5 mM thymidine for 16 h followed by extensive wash and release into normal growth medium for 10 h to obtain cells in G_2_/M. The cell cycle stage was monitored by staining with propidium iodide (10 μg/ml), followed by flow cytometric analysis performed on a BD^™^ LSR II (BD biosciences) equipped with FlowJo software (version vX 0.7).

### Quantitative PCR (qPCR) analysis

Total RNA was extracted from cultured cells with RNeasy Mini kit (Qiagen), with the use of QIAshredder spin column for homogenization and an on-column DNase digestion. 2 μg of the total RNA was reversely transcribed using M-MLV reverse transcriptase enzyme (Promega). The cDNA obtained was analysed quantitatively using Power SYBR Green PCR Master Mix (Applied Biosystems) on an ABI7300 Real-time PCR system. Primers used are listed in Table 2. Cycling conditions were 95 °C for 15 min, 40 cycles of 15 s at 94 °C, 30 s at 55 °C and 30 s at 72 °C. Ct values were generated using default analysis settings. Relative quantification (RQ) was calculated using 2 ^–ΔΔCT^ method.

**Table 1:** Pre-treatment characteristics of patients

*Pre-treatment characteristics of patients, n = 21*	
Characteristic	
Age	
Median (yr)	61 (32–77)
Sex-no. (%)	
Male	16 (76.2 %)
Female	5 (23.8 %)
ECOG performance status- no. (%)	
0	18 (85.7 %)
1	3 (14.3 %)
Clinical staging (EUS/CT staging)	
T1	0 (0 %)
T2	2 (9.5 %)
T3	17 (81.0 %)
T4	2 (9.5 %)
N0	7 (33.3 %)
N+	14 (66.7 %)
Histology grade	
Moderately differentiated	3 (14.3 %)
Poorly differentiated	18 (85.7 %)

### Bisulphite conversion and methylation-specific polymerase chain reaction (MSP)

DNA was extracted from the gastric carcinoma cell lines with Puregene^™^ DNA Isolation Kit (Gentra Systems). 500 ng of the cell line DNA, positive control DNA (CpGenome Universal Methylated DNA, Chemicon) and negative control Human Sperm DNA (HsD) were used for bisulphate conversion using EZ DNA Methylation-Gold™ Kit (Zymo Research Corporation) as per manufacturer’s protocol. Primers used for MSP reactions are listed in Table 3. PCR was performed by preheating at 94 °C for 5 min, then 40 cycles of denaturation at 94 °C for 30 s, annealing at 56 °C/56 °C for 60 s for methylated/unmethylated GTSE1, and extension at 72 °C for 60 s, followed by a final 7 min extension at 72 °C. The PCR products were separated on a 2 % agarose gel.

**Table 2:** RT PCR primer sequences used to detect GTSE1 mRNA expression

RT-PCR primers	Sequence
GTSE1 forward	5-GCC CCG GGT GCT GTC AAT GT-3
GTSE1 reverse	5-GCC CAC TGC TGG GGA TGT GC-3
GAPDH forward	5′-TGA AGG TCG GAG TCA ACG GAT TTG GT-3
GAPDH reverse	5′-CAT GTG GGC CAT GAG GTC CAC CAC-3′

**Table 3:** MSP primer sequences used to detect GTSE1 DNA methylation

Methylation/ Unmethylation primers	Sequence
Methylated GTSE1 forward	5′-GTA GTG CGT ATG CGT ATT GGA C−3′
Methylated GTSE1 reverse	5′-GCG AAT TAC CGA TTA ATC GAT−3′
Unmethylated GTSE1 forward	5′-AGT AGT GTG TAT GTG TAT TGG ATG−3′
Unmethylated GTSE1 reverse	5′-AAA ACA CAA ATT ACC AAT TAA TCA AT−3′

### Small hairpin (sh) RNA transfection

Human GTSE1 ‘SureSilencing shRNA’ plasmids were purchased from Qiagen. AZ521 cells (1 × 10e5 cells/well) were plated in 6-well plates and grown in 2 ml RPMI-1640 medium with 10 % FBS. Transfections of GTSE1 shRNA and control vectors were performed using X-treme gene transfection reagent (Roche) according to the manufacturer’s instructions. Independent colonies were isolated by ring cloning, and expanded in 1000 μg/ml neomycin. Cell lysates were collected and GTSE1 expression was detected by western blotting.

### MTS- cell viability assay

To assess the chemosensitivity of tumor cells to cisplatin, cell viability was measured by MTS (Colorimetric CellTiter 96 Aqueous One Solution Cell Proliferation Assay) (Promega). Cell suspension was cultured in 96-well flat-bottomed microtiter plates at seeding density of 2 × 10e3 cells/well and incubated overnight. Drug treatments were carried out at a dilution range of 0.01-1000 μM cisplatin. Microtiter wells containing tumour cells with no drug treatments were used as controls, and wells containing complete medium were used as blank controls. Cells were incubated for 72 h before the addition of MTS solution (1 mg/mL per well) and absorbance was read at 550 nm using a spectrophotometric microplate reader (Bio-Rad). The percentage cell viability at different drug concentrations was calculated as the inhibition rate of (mean absorbance of treated wells/mean absorbance of control wells) × 100 %. IC50 was calculated by GraphPad Prism v4.0 (GraphPad Software, Inc).

### Cell proliferation (BrdU) assay

Cells were seeded on to 96-well plates at a density of 2 × 10^3^ cells/well. Cell proliferation was measured using the BrdU proliferation assay (Roche) at 12, 24 and 48 h according to the manufacturer’s protocol. The absorbance values are directly correlated to the amount of DNA synthesis.

### Colony formation assay

The ability for colony formation at low cell density was determined by plating 1 × 10^3^ cells/well onto a 6-well plate and then culturing for 7 days. Cells were subsequently stained with 0.5 % crystal violet in 30 % ethanol and 3 % formaldehyde for 10 min at room temperature. Stained colonies were counted under the microscope by selecting a total of five random fields per sample.

### Apoptosis assay

Apoptosis was detected by Annexin V-FITC (fluorescein isothiocyanate) kit (BD Pharmingen) according to manufacturer’s instructions. Briefly, the cells (1 × 10^5^ cells/ml) were grown to 80 % confluency in 25 cm^2^ flasks in F12K supplemented with 10 % fetal calf serum. After 24 h of incubation with or without drug, cells were harvested, washed thrice with cold PBS and resuspended in 1× binding buffer. An aliquot of 100 μl of the cell suspension was transferred into a microfuge tube and mixed with equal volumes (5 μl) of Annexin V- FITC and Propidium Iodide (PI). The cells were gently vortexed and incubated for 15 min at 37 °C in the dark, before the addition of 400 μl of 1X binding buffer in each tube. The cell samples were then analysed using a flow cytometer BD^™^ LSR II (BD biosciences) equipped with FlowJo software (version vX 0.7).

### Protein extraction and western blot analysis

Cells were washed with ice cold PBS and whole cell lysates were prepared using CelLytic buffer (Sigma-Aldrich). The cytoplasmic and nuclear protein fractions were extracted using NE-PER Nuclear and Cytoplasmic Extraction kit (Thermo Scientific) as per manufacturer’s instructions. 20 μg of protein was electrophoretically separated on 12 % SDS-PAGE. Western blots were performed with the primary antibodies: anti-GTSE1 (Santa Cruz biotechnology), anti-p53, anti-Bax, anti-p21 Waf1/Cip1 (12D1), anti-GAPDH (Cell Signaling) and the corresponding secondary antibodies: anti-rabbit/mouse IgG, HRP-Linked (Cell Signaling). The signals were visualized by ECL reagent (Amersham^™^ ECL Plus Western Blotting Detection System; GE Healthcare), followed by exposure to chemiluminescence film (Amersham Hyperfilm^™^ ECL; GE Healthcare). Immunoblot analyses were repeated twice for each protein tested.

### Statistical analysis

Two-tailed student’s *t*-test was used for differential comparison between two groups. *P* value <0.05 was considered statistically significant. Differential methylation analyses between 2 groups were performed using the statistical package in R (www.r-project.org).

## Results

### GTSE1 is highly expressed in primary gastric tumours and correlates with hypomethylation in gastric cancer cells

**Fig. 1:**
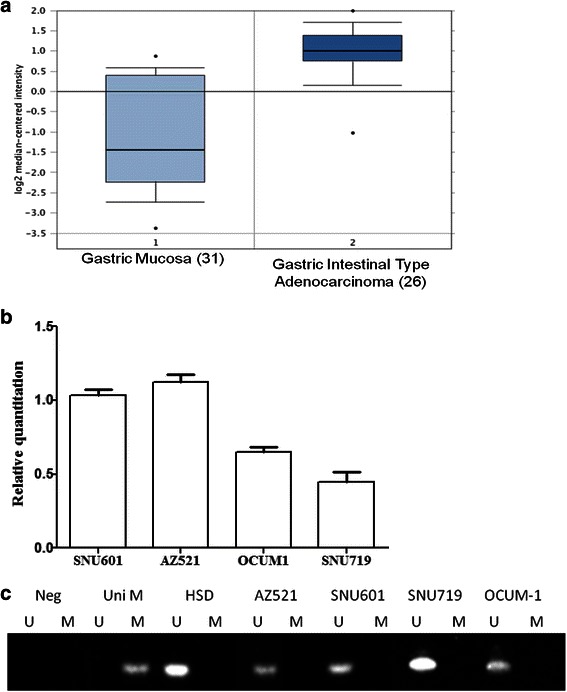
**a** GTSE1 Log2 transformed expression level. Fold change of each sample between tumor tissues and non-tumorous tissues in microarray from GEO in gastric cancer (GSE13911) is shown. **b** The mRNA expression of GTSE1 in 4 gastric cancer cells detected by qPCR. The gene expression of GTSE1 was normalised relative to GAPDH as internal control. The p value shown for the average data was computed using a paired two-tailed Student t test (n = 3). Error bars denote standard deviations. **c** DNA promoter methylation status of MAGE-A1 in 4 gastric cell lines by MSP. UniM: Universal methylated DNA is the control sample for methylation; HSD: human sperm DNA is the control sample for unmethylation. M: methylation; U: unmethylation

In order to verify the expression level of GTSE1 mRNA, we analyzed the public microarray database, Gene Expression Omnibus (Gastric cancer: a, GSE13911., b, GSE27242). GTSE1 was up-regulated in both datasets with a fold change of 4.07 and 3.34 and *t*-test p-value 7.37E-10 and 1.72E-6 respectively. Figure 1a shows the log2 transformed fold change of GTSE1 mRNA level in tumor tissue versus non-tumor in GSE13911. These findings were further supported by investigating GTSE1 mRNA expression in gastric cancer cell lines. As shown in Fig. 1b, GTSE1 expression in 4 gastric cancer cell lines- AZ521, SNU60, OCUM-1 and SNU719 was analysed by qPCR. All the cell lines showed detectable expression of GTSE1 mRNA, among which AZ521 showed a relatively high GTSE1 expression, whereas SNU 719 showed a relatively low expression. Furthermore, the association between expression and methylation status was determined by MSP (Fig. 1c). A 100 % (4/4) concordance was observed between GTSE1 hypomethylation and expression as all the cell lines were unmethylated for GTSE1.

### Differential methylation of GTSE1 correlates with treatment response to DCX and Cispaltin sensitivity

**Fig. 2:**
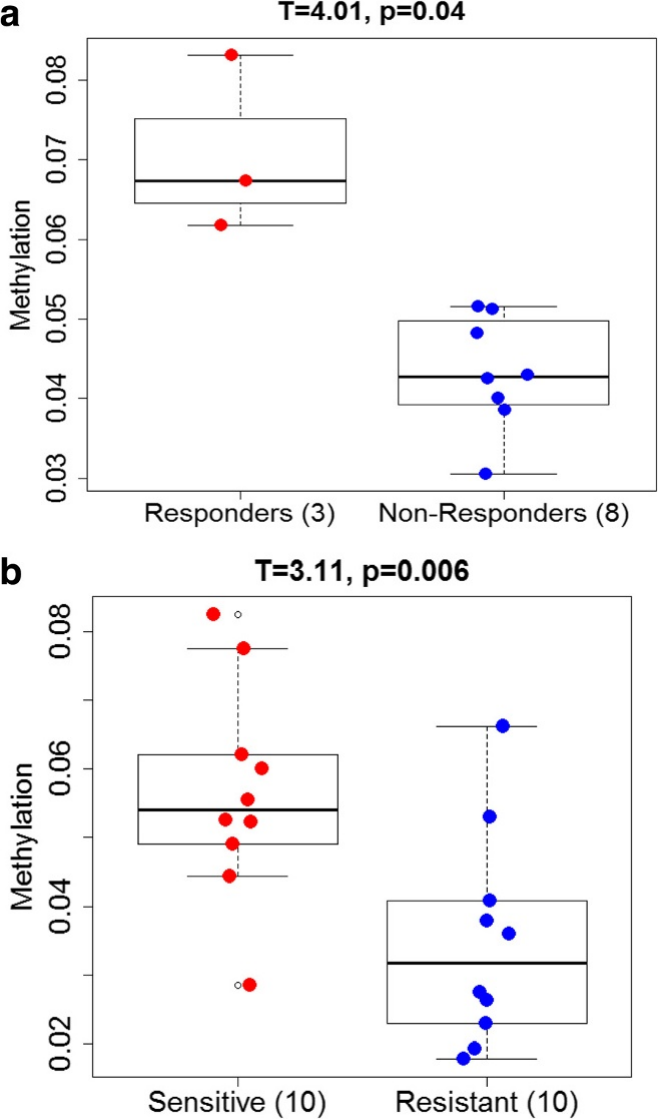
**a** GTSE1 methylation in gastric cancer patients. Patient samples were classified as responders or non-responders by pathological response (Responders: complete response or near-complete response; Non-responders: partial response or others). **b** GTSE1 methylation in gastric cell lines. Differential methylation analyses were carried out between the top 10 cisplatin resistant and top 10 cisplatin sensitive groups

Methylation profiling of tissue samples obtained from 11 GC patients before receiving DCX treatment depicted hypomethylation of GTSE1 (range: 0.03-0.08). GTSE1 was shown to be differentially methylated in GC patients who are responders to DCX combinational chemotherapy. Statistical analyses revealed that GC patients who were classified as responders (n = 3) by pathological response have significantly higher GTSE1 methylation (T = 4.01, *p* = 0.04) as compared to non-responders (n = 8) (Fig. 2a). Similarly, methylation profiling of 39 GC cell lines also suggested hypomethylation of GTSE1 (range: 0.01-0.08). Statistical analyses performed on the top 10 cisplatin-sensitive and top 10 cisplatin-resistant cell lines revealed significantly higher GTSE1 methylation in the sensitive cell lines as compared to the resistant ones (Fig. 2b: T = 3.11, *p* = 0.006). Conversely, no association was found between GTSE1 methylation and drug sensitivity of gastric cancer cells to docetaxel and 5FU (Additional file 1: Figure S2).

### GTSE1 knockdown enhanced cisplatin sensitivity in gastric cancer cells

**Fig. 3:**
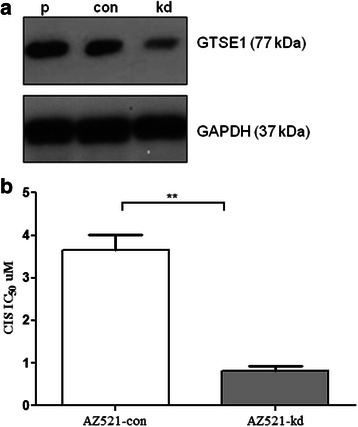
**a** Immunoblot of GTSE1 expression in AZ521 cells. p: parental cell lines, con: scrambled controls, kd: knockdown variants of GTSE1. At least two independent experiments were preformed for each cell line. **b** MTS assay to determine IC50 of cisplatin (CIS) in AZ521-cont and its GTSE1 knockdown variant cell line AZ521-kd. Values represent average of two independent experiments and error bars denote standard deviations

The association between GTSE1 expression and chemo-resistance to cisplatin was defined. GTSE1 was stably knocked-down in AZ521 cells by transfection with targeted shRNA’s. Transfected variants of AZ521 cells (AZ521-kd) showed a significant reduction in GTSE1 protein expression compared to the cells transfected with the scrambled control shRNA (AZ521-cont) and the parental cell lines (AZ521-p) (Fig. 3a). To detect the role of GTSE1 in chemo resistance, the dose response of AZ521 cells to cisplatin treatment was analysed. Interestingly, loss of GTSE-1 expression contributed to enhanced sensitivity to cisplatin treatment as shown by a ~5 fold decrease in IC50 values of AZ521-kd cells (0.8 μM) compared to the parental counterpart (3.7 μm) (*p* = 0.004) (Fig. 3b). The study suggests GTSE-1 to play a major role in cisplatin resistance in gastric cancer cells. The drug resistance showed by GTSE1 expressing cells appeared highly specific to cisplatin treatment, as similar dose response studies using docetaxel and 5FU in GTSE1 knocked-down cells yielded no significant variations in IC50 (Additional file 1: Figure S3).

### GTSE1 knock down enhanced cisplatin cytotoxicity by causing a reduction in proliferation and colony formation of gastric cancer cells

**Fig. 4:**
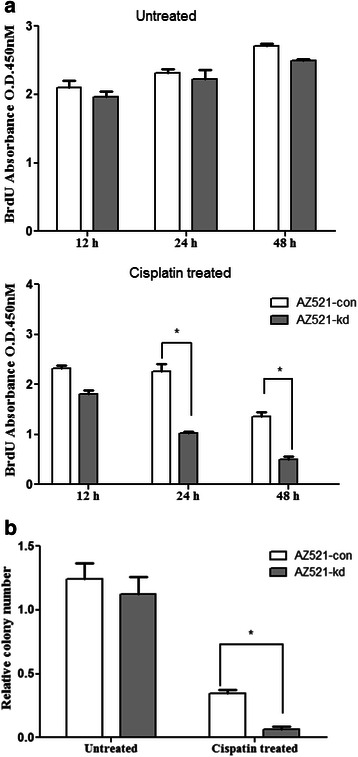
**a** Cell proliferation assay. Proliferation rate of AZ521-con and AZ521-kd cells as evaluated with the BrdU assay at 12, 24 and 48 h post cisplatin treatment. Untreated cells served as experimental control. Results are representative of two independent experiments and error bars indicate standard deviations. **b** Clonogenic survival assay. The colony formation ability of AZ521-con and AZ521-kd cells were measured after cisplatin treatment. Untreated cells served as experimental control. Results are representative of two independent experiments and error bars indicate standard deviations

The cytotoxic effects of cisplatin on the proliferation of AZ521 cells were measured by BrdU cell proliferation assay (Fig. 4a). Cells pre-treated with cisplatin for 12, 24 and 48 h showed a time dependent reduction in overall proliferation as compared to the untreated cells. Interestingly, GTSE1 knock down resulted in a greater reduction in cell proliferation as AZ521-kd cells exhibited a sharp decline in proliferation within 24 h of cisplatin treatment (*p* = 0.01). The results were more significant at 48 h of drug treatment, as observed by a fivefold decrease in proliferative cells (*p* = 0.003). To further confirm the enhanced cytototoxic effects of cisplatin in GTSE1 knock down cells, a clonogenic survival assay was performed. Although cisplatin treatment induced an inhibitory effect in the colony formation ability of both AZ521-con and AZ521-kd cells, the decrease of colony formation in GTSE1 knocked-down cells appeared to be more drastic and highly significant (*p* = 0.002) as compared to the control cells (Figure 4b). Taken together, our findings suggest a role of GTSE1 in enhancing the growth and survivability of gastric cancer cells where it acts antagonistic to the cellular sensitivity towards cisplatin.

### Cisplatin cytotoxity upregulates GTSE1 expression and induces its nuclear translocation

**Fig. 5:**
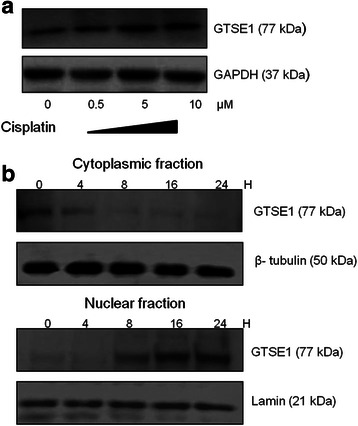
GTSE1 expression in cisplatin treated AZ521 cells (**a**) AZ521 cells were treated with varying concentrations of cisplatin. The whole cell lysates were extracted post 24 h of treatment and GTSE expression was detected by western blotting. GAPDH served as loading control (**b**) GTSE1 sub cellular localization: AZ521 cells were treated with cisplatin for various time intervals and subcellular fractions were extracted and blotted for GTSE1 expression. Lamin and tubulin served as loading controls for nuclear and cytoplasmic fractions respectively

Previous studies have shown an increase in GTSE1 expression in response to DNA damage insults (Monte *et al.*, [11], [14]). Consistently, the present study observed a dose dependent up regulation of GTSE1 expression in cisplatin treated AZ521 cells (Fig. 5a). The expression levels peaked at close to IC50 concentration of cisplatin. Moreover, a change in GTSE1 subcellular localization was also observed as cisplatin treatment induced the translocation of GTSE1 from the cytoplasm to the nucleus in a time dependent manner. As shown in Fig. 5b GTSE1 expression appeared predominantly cytoplasmic in untreated AZ521 cells. Upon 8 h of cisplatin treatment, a significant reduction in GTSE1 expression was observed in the cytoplasmic fraction whereas a corresponding increase in expression was seen in the nuclear fractions. These findings are in accordance with and add further credence to the studies by Monte *et al.* [14] that showed nuclear accumulation of GTSE1 during DNA damage.

### GTSE1 expression down regulates cellular apoptosis by repressing p53 signaling

**Fig. 6:**
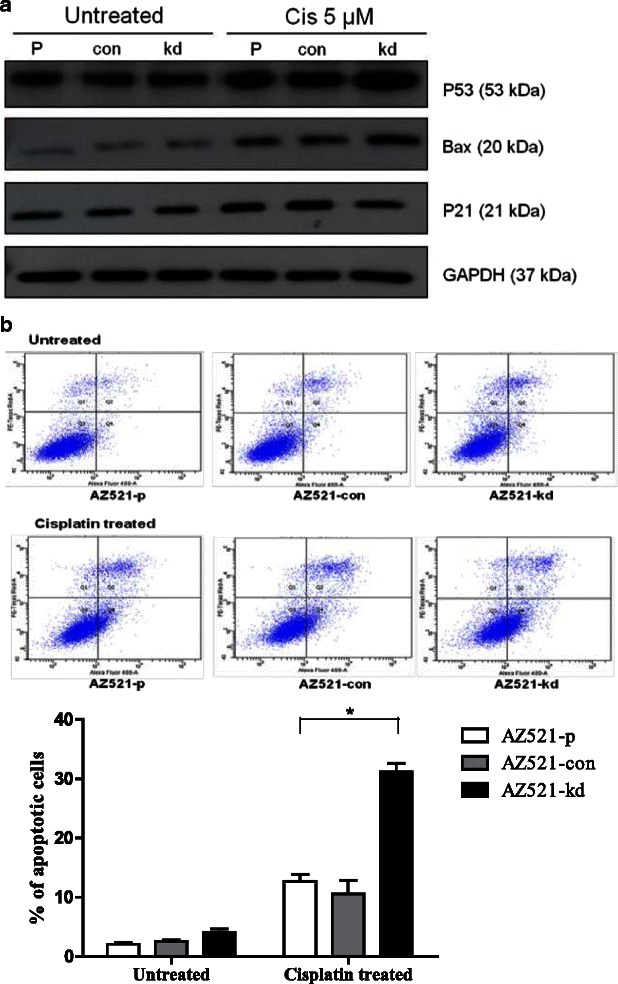
Role of GTSE1 in cisplatin induced apoptosis. **a** AZ521-p, AZ521-con and AZ521-kd cells were treated with cisplatin (5 μm) for 24 h and expression of pro-apototic p53 and its downstream effectors bax and casp-3 were detected by western blotting. Untreated cells served as experimental control. GAPDH served as internal control (**b**) The induction of apoptosis in AZ521-p, AZ521-con and AZ521-kd as detected by flow cytometry using Annexin V-FITC apoptosis assay. The percentage of apoptotic cells (lower right quadrant) was calculated. Data are presented as the mean values of the florescent intensities from two independent experiments and error bars standard deviations (SD)

Studies have shown that p53 expression is up regulated upon chemotherapeutic stress and GTSE1 was previously characterised as a negative regulator of p53 (Scolz *et al.*, [15]; Zhan *et al.*, 2014). This prompted us to further investigate the mechanism of cisplatin sensitivity in GTSE1 knocked-down gastric cancer cells. Treatment of AZ521 cells with cisplatin caused an up regulation of p53 expression and its downstream effector bax. Interestingly, knock down of GTSE1 expression lead to a further increase in p53 and bax expression levels. This increase in expression in GTSE1 knock down cells appeared specific to cisplatin cytotoxity as untreated cells did not show any corresponding variation in p53 and bax levels (Fig. 6a). Treatment of AZ521 cells with cisplatin also resulted in caspase 3 cleavage suggesting induction of apoptosis (Additional file 1: Figure S4).On the other hand, a down regulation in p21 levels was observed in GTSE1 knocked-down cells. This down regulation of p21 converges with the previous shown functionality of GTSE1, where it prevents proteosome dependent degradation of p21. Notably, the biological consequence of p53 upregulation in GTSE1 knocked-down cells is also reflected by a significant up regulation in cisplatin induced apoptosis. As shown in Fig. 6b, GTSE1 knock down enhanced the sensitivity of AZ521 cells to cisplatin treatment resulting in a >50 % increase in apoptosis compared to the parental cells (*p* = 0.010).

## Discussion

The use of modern genomic, proteomic and functional analytical techniques has resulted in a substantial increase in our ability to identify novel genes and signaling networks that are involved in determining the responsiveness of tumors to a particular drug treatment. The mechanisms of resistance to ‘classical’ cytotoxic chemotherapeutics and to therapies that are designed to be selective for specific molecular targets share many features, such as alterations in the drug target, activation of pro-survival pathways and ineffective induction of cell death [9]. Pre-clinical and clinical studies have already shown that combining information from more than one molecular biomarker increases our ability to predict tumour drug response [19]. Hence, understanding how tumors evolve on a molecular level to overcome the cytotoxic effects of chemotherapy is a critical step in developing therapeutic approaches that will prevent or overcome chemoresistance [20].

Alterations in gene expression profiles and epigenetic changes are known to be early events in the multi-steps of carcinogenesis [21]. On an epigenome-wide basis, multiple DNA methylation changes in the cancer methylome have been reported to occur during the acquisition of drug resistance [22]. Here, we explored the contributory role of cell cycle dependent protein GTSE1 in gastric cancer chemoresistance. GTSE1 is highly expressed in cancers and was shown to be clinically associated with drug resistance [18]. Analysis of public gene array databases showed high GTSE1 expression in gastric cancer [23–25]. Since aberrant DNA methylation as a marker of platinum-resistance was shown in lung and ovarian cancer cell lines [26, 27]; we examined the methylation status of GTSE1 in gastric cancer patients who underwent neoadjuvant DCX-combinational chemo therapy. Although GTSE1 appeared hypomethylated in both responders and non responders of DCX treatment, a clear distinction could be seen between the two groups wherein responders showed a higher methylation index. However, the current study was unable to show any significant survival benefit of GTSE1 methylation in gastric cancer patients. This could be due to the small sample size and thus hinders evaluation of GTSE1 methylation as a predictive biomarker for DCX treatment response. Consistently, GTSE1 was shown to be hypomethylated in gastric cancer cell lines. All hypomethytlated cell lines showed significant expression levels of GTSE1. Intriguingly, cell lines that are sensitive to cisplatin treatment showed a significantly different methylation index for GTSE1. Since, the predominant model of regulation of gene expression underscores an inverse correlation between DNA methylation and expression [28], the higher methylation index of GTSE1 in DCX responders and in cisplatin sensitive cell lines could be suggestive of its lower expression levels. However, this study did not investigate this correlation in the DCX patient cohort. Hence, clinical implications of GTSE1 methylation status needs further investigation regarding the possible rationales raised in this study

Gene expression profiling in cancer have provided substantial information on the oncogenic potential of genes controlling essential pathways and other cellular events. Cell proliferation and growth inhibition are tightly regulated processes which are key in the maintenance of normal cell growth homeostasis and viability [29]. However deregulation of these processes occur during tumor development and is often contributory to drug resistance. The present study demonstrated a major role of GTSE1 in conferring cisplatin resistance as knock down of GTSE1 expression in gastric cancer cells enhanced cisplatin sensitivity. Moreover, cisplatin treatment induced a dose dependent upregulation as well as nuclear translocation of GTSE1 in gastric cancer cells. The increase in expression and change in membrane localization of GTSE1 falls in line with a previous study that showed up regulation and nuclear import of this protein in response to various DNA- damaging agents [14]. The nuclear translocation of GTSE1 is also suggestive of the cellular response to cisplatin induced DNA damage stress, where its expression counteracts with drug sensitivity. Enhanced proliferation and colony formation ability of cells are well associated with malignant transformation in many cancers [30]. Although anti-cancer therapy targets apoptosis of hyper-proliferative cells, its efficacy may vary according to cancers and its molecular sub-types. In our study, loss of GTSE1 expression enhanced the anti-proliferative and growth-inhibitory effects of cisplatin in gastric cancer cells. Of note, the decrease in cell proliferation and colony formation observed in GTSE1 knocked-down cells is also suggestive of its tumorogenic potential that could be utilized in targeted therapies in gastric cancer. Consistent with these observations, expression of GTSE1 was found inhibitory to cisplatin cytotoxicity as its knock down lead to a two-fold increase in apoptosis of gastric cancer cells. This increase in apoptosis could well be explained by the observed increase of p53 expression in GTSE1 knocked-down cells. P53 is a potent inhibitor of cell growth and its function is tightly controlled to allow normal growth development [31]. In response to DNA damage insults, p53 induces cell cycle arrest and activates the intrinsic apoptotic signaling pathway [32]. P53 up regulation also resulted in activation of its downstream effectors- bax. This could attribute to chromatin condensation, DNA fragmentation and finally apoptosis [33]. Hence, up regulation of p53 and its downstream effectors in cisplatin treated cells can be inferred as a cellular response to DNA damage. Therefore, by identifying a reduced expression profile of p53 in normal cells against GTSE1 knocked-down cells, our study demonstrates a role of GTSE1 in attenuating p53 mediated apoptotic response in cisplatin treated gastric cancer cells. However, we did not see any significant difference in caspase 3 activation in GTSE1 knock down cells upon cisplatin treatment, as compared to its parental counterparts (Additional file 1: Figure S4). Our findings suggest that GTSE1 mediates a caspase 3 independent cascade of apoptotic repression in cisplatin treated gastric cancer cells. This is consistent with previous studies by and Cui *et al.* and Hu *et al.* that have shown a p53 dependent but caspase 3 independent mechanism of apoptosis [34, 35]. In addition, GTSE1 expression was also seen to be associated with high expression of p21 as knockdown of GTSE1 caused a reduction in p21 turn over. This falls in agreement with studies by Bublik *et al.* that showed GTSE1 as a regulator of p21 stability by protecting it from proteosome-dependent degradationEarlier, the association of GTSE1 with p21 was shown to modulate cellular response to paclitaxel induced apoptosis in cervical cancer cells [36]. Moreover, high levels of p21 in cancers have been linked to poor prognosis [37]. Thus, by oppositely regulating p53 and p21, GTSE1 protein may display a combined role in promoting cell survival by shifting the equilibrium of p53 response from apoptosis to survival [23]. Hence, targeting GTSE1 in cancer therapy could enhance a p53 mediated pro-apoptotic response with a reflective decrease in p21 induced cell cycle arrest.

## Conclusion

Individualisation of therapy according to the molecular phenotype of tumour and patient could dramatically increase the effectiveness of chemotherapy. The study presented here emphasizes the predictive value of GTSE1 as a biomarker for cisplatin resistance in gastric cancer. Although previous studies in lung cancer patients did not show any correlation of GTSE1 with clinical data, the drug sensitivity profile and down-regulation of p53 induced apoptotic signaling in GTSE1 knocked-down gastric cancer cells is intriguing and warrants an in depth analysis of its clinical significance. Future studies should utilize animal models to further explore the therapeutic utility of GTSE1 in gastric cancer. Nevertheless, our study identifies a previously uncharacterized role of GTSE1 in conferring cisplatin resistance and presents an additional avenue for future therapeutic intervention and patient stratification in gastric cancer.

## Additional file

Additional file 1: Table S1. Surgical results. **Figure S1.** Radiological response after 2 cycles of chemotherapy (n = 17). **Figure S2.** GTSE1 methylation in gastric cell lines (a) Differential methylation analyses were carried out between the top docetaxel resistant and docetaxel sensitive groups. (b) Differential methylation analyses were carried out between the top 5FU resistant and 5FU sensitive groups. **Figure S3.** MTS assay to determine IC50 of (a) docetaxel (DOC) and (b) 5FU in AZ521-cont and its GTSE1 knockdown variant cell line AZ521-kd. Values represent average of two independent experiments and error bars denote standard deviations. **Figure S4.** Caspase 3 cleavage in cisplatin treated AZ521 cells. a) AZ521-p, AZ521-con and AZ521-kd cells were treated with cisplatin (5 μm) for 24 h and caspase 3 expression was detected by western blotting. Untreated cells served as experimental control. GAPDH served as loading control. (DOC 710 kb)
