# Supplementary material for: GTSE1 expression represses apoptotic signaling and confers cisplatin resistance in gastric cancer cells
Source: BMC Cancer. 2015 Jul 25;15:550. doi: 10.1186/s12885-015-1550-0 (PMC4514980; doi:10.1186/s12885-015-1550-0)
Supplement: Additional file 1: Table S1. — Surgical results. Figure S1. Radiological response after 2 cycles of chemotherapy (n = 17). Figure S2. GTSE1 methylation in gastric cell lines (a) Differential methylation analyses were carried out between the top docetaxel resistant and docetaxel sensitive groups. (b) Differential methylation analyses were carried out between the top 5FU resistant and 5FU sensitive groups. Figure S3. MTS assay to determine IC50 of (a) docetaxel (DOC) and (b) 5FU in AZ521-cont and its GTSE1 knockdown variant cell line AZ521-kd. Values represent average of two independent experiments and error bars denote standard deviations. Figure S4. Caspase 3 cleavage in cisplatin treated AZ521 cells. a) AZ521-p, AZ521-con and AZ521-kd cells were treated with cisplatin (5 μm) for 24 h and caspase 3 expression was detected by western blotting. Untreated cells served as experimental control. GAPDH served as loading control. (DOC 710 kb) [file 12885_2015_1550_MOESM1_ESM.doc]

| ***Pre-treatment characteristics of patients, n=21*** | |
| --- | --- |
| ***Characteristic*** | |
| **Age** | |
| Median (yr) | 61 (32-77) |
| **Sex-no. (%)** | |
| Male | 16 (76.2%) |
| Female | 5 (23.8%) |
| **ECOG performance status- no. (%)** | |
| 0 | 18 (85.7%) |
| 1 | 3 (14.3%) |
| **Clinical staging (EUS/CT staging)** | |
| T1 | 0 (0%) |
| T2 | 2 (9.5%) |
| T3 | 17 (81.0%) |
| T4 | 2 (9.5%) |
| N0 | 7 (33.3%) |
| N+ | 14 (66.7%) |
| **Histology grade** | |
| Moderately differentiated | 3 (14.3%) |
| Poorly differentiated | 18 (85.7%) |

**Table 1.**
